# Case report: Green nail syndrome in an epidemic prevention volunteer during the outbreak of the Omicron in Shanghai

**DOI:** 10.3389/fpubh.2022.1009517

**Published:** 2022-09-20

**Authors:** Qian Yu, Sheng Hu, Wei Li, Lianjuan Yang

**Affiliations:** ^1^Department of Medical Mycology, Shanghai Dermatology Hospital, Tongji University School of Medicine, Shanghai, China; ^2^Central Laboratory, Shanghai Dermatology Hospital, Tongji University School of Medicine, Shanghai, China; ^3^Department of Medical Cosmetology, Shanghai Dermatology Hospital, Tongji University School of Medicine, Shanghai, China

**Keywords:** green nail syndrome, *Pseudomonas aeruginosa*, protective clothing, epidemic prevention volunteer, Omicron

## Abstract

Green nail syndrome (GNS) is an infectious disorder characterized by greenish discoloration of the nail plate. *Pseudomonas aeruginosa* is the most common organism that causes GNS. It is an opportunistic human pathogen that preferentially colonizes moist environments, and thus, it usually affects patients with a history of prolonged exposure to moist environments. Here, we describe a case of GNS in an epidemic prevention volunteer that was caused by wearing personal protective equipment for prolonged durations. The case was reported during the outbreak caused by the SARS-CoV-2 Omicron variant in Shanghai. After receiving information about his condition and proper treatment, the patient was cured.

## Introduction

Green nail syndrome (GNS), also known as chloronychia, is an infectious disorder characterized by greenish discoloration of the nail plate (1). *Pseudomonas aeruginosa* is the most common causative pathogen (2). GNS may be viewed as an occupational disease amongst homemakers, barbers, dishwashers, and medical personnel (3).

It is well-known today that various skin disorders are induced by the use of personal protective equipment (PPE) such as facemasks and gloves (4). Here, we describe a case of GNS in an epidemic prevention volunteer caused by wearing PPE for prolonged durations.

## Case report

**Figure 1:**
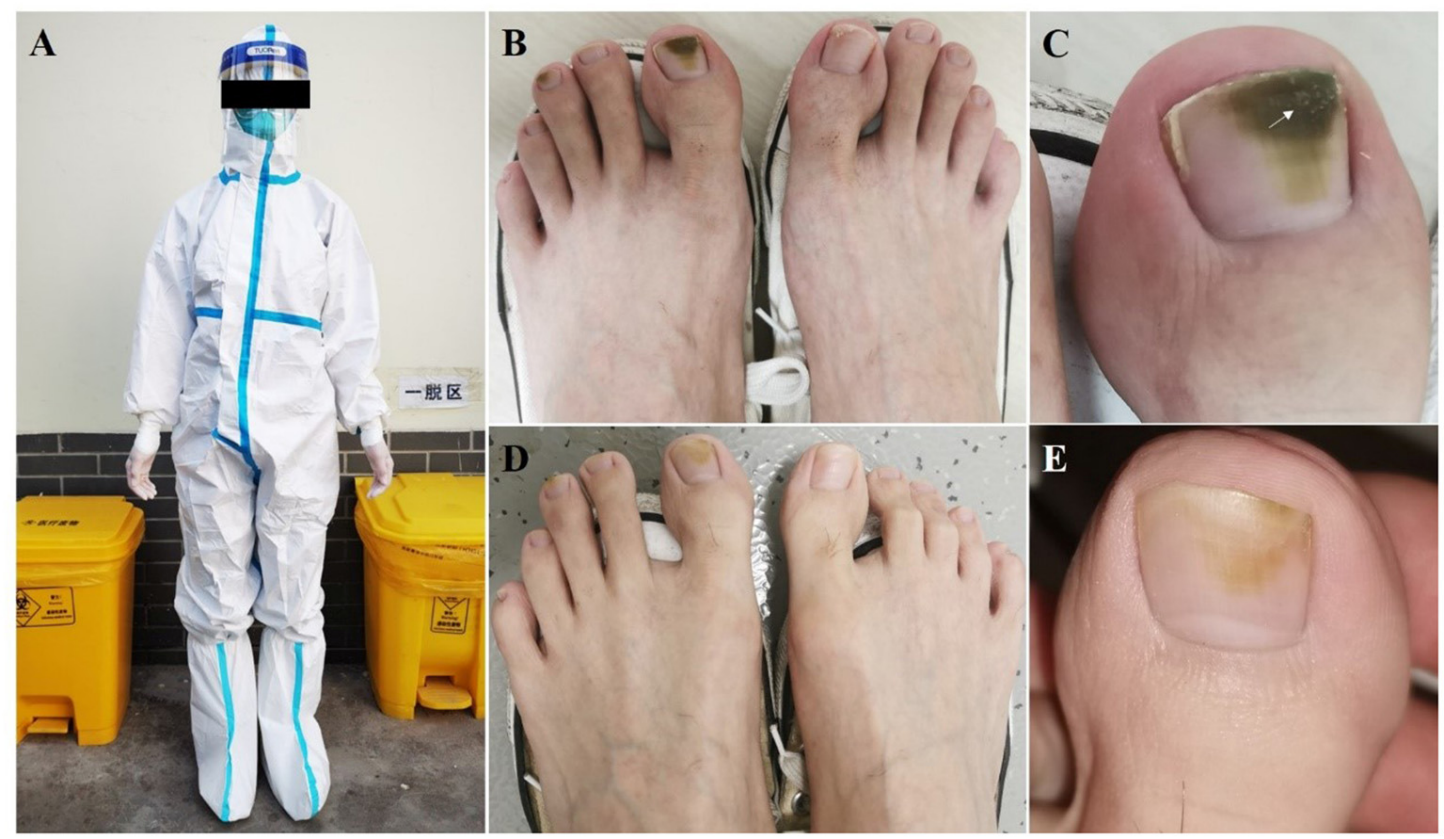
Clinical Images. **(A)** Epidemic prevention volunteer wearing personal protective equipment. **(B,C)** Greenish black discoloration of the middle and distal parts of the first and third toenails on the left foot; white scale (white arrow) on the distal part of the big toenail plate. **(D,E)** At the 2-month follow-up, the greenish discoloration had faded significantly.

A 34-year-old otherwise healthy male presented with asymptomatic, greenish discoloration of the nail plate that had set in over a period of 4 weeks. The discoloration began at the distal margin extending to the middle of the nail plate. The patient did not report any history of trauma or onychosis. Before the lesions appeared, he was an epidemic prevention volunteer who wore PPE (Figure 1A) for at least 10 h daily. He washed his feet for an average of two to three times per shift during the outbreak of the Omicron variant of severe acute respiratory syndrome coronavirus (SARS-CoV-2) in Shanghai.

Physical examination revealed greenish black discoloration from the middle to distal parts of the first and third toenails of the left foot (Figure 1B). A white scale was observed on the distal part of the big toenail plate on the left foot (Figure 1C). Neither onycholysis nor paronychia was observed around the affected nails. Direct fluorescence microscopy and fungal culture of nail scrapings were negative; however, bacterial culture of the nail scrapings was positive for *P. aeruginosa*. Subsequently, drug sensitivity tests were performed, which revealed sensitivity to fluoroquinolones including ciprofloxacin and levofloxacin.

GNS was diagnosed based on these findings, and the patient was instructed to stop wearing PPE, to wash his feet daily, and to keep his feet dry. Based on previous literature (5, 6) and drug sensitivity tests, he was prescribed treatment that included the application of topical nadifloxacin in combination with acetic acid twice daily. At the 2-month follow-up, discoloration of the nail was significantly reduced (Figures 1D,E).

## Discussion

GNS usually presents with a triad of green discoloration of the nail plate, proximal paronychia, and distal onycholysis. The characteristic greenish discoloration of the nail is due to pyoverdine and pyocyanin that are pigments produced by *P. aeruginosa* (7). This organism is an opportunistic human pathogen that preferentially colonizes moist environments and, thus, usually affects patients with a history of prolonged exposure to water (8). When the integumentary barrier of the nail is impaired owing to any one of the various causes, coupled with a moist environment, it could promote the occurrence of GNS. GNS is also observed in patients with nail diseases such as onycholysis, onychotillomania, chronic paronychia, psoriasis (9), and onychomycosis (6).

As an epidemic prevention volunteer, our patient engaged in activities such as disinfection and sterilization in the community, delivery of daily necessities to residents, transportation of patients with COVID-19. He walked for an extended period daily and experienced nail damage due to repeated friction and extrusion of nail plates within his shoes, which led to *P. Aeruginosa* colonization. Moreover, the patient had to wear PPE for 10 h daily, which led to a moist and occlusive environment where *P. Aeruginosa* thrived. Recently, an association between nail art and GNS has been reported, possibly because the patient from the report removed the gel nail polish by buffing off the nail plate, which likely resulted in microtrauma and disrupted the nail barrier, triggering GNS (10). Likewise, in our patient, *P. Aeruginosa* colonization transformed into an infection significant enough to result in GNS. Later, when our patient complied with health education and treatment protocols, we observed a positive response to treatment.

This study has some limitations. First, our patient received topical nadifloxacin combined with acetic acid. This approach was based on previous literature and drug sensitivity tests. The optimal treatment regimen for GNS should be explored. Second, this is a single case, the incidence of GNS in epidemic prevention volunteer should be further investigated.

In conclusion, wearing PPE for extended periods contributes to a humid environment near the feet and could potentially lead to the development of GNS. Epidemic prevention volunteers actively serve community residents; meanwhile, they must pay attention to self-care, especially regarding PPE-related dermatological diseases.

## Data availability statement

The original contributions presented in the study are included in the article/supplementary material, further inquiries can be directed to the corresponding author/s.

## Author contributions

QY and SH: design and draft of the work and analysis, acquisition, and interpretation of data. WL and LY: study design, revision, and finalization of the manuscript. All authors contributed to the article and approved the submitted version.

## Funding

This work was supported by the Science and Technology Commission of Shanghai Municipality (Grant Number 20Y11905600) and the Shanghai Municipal Commission of Health and Family Planning (Grant Number 20194Y0337).

## Conflict of interest

The authors declare that the research was conducted in the absence of any commercial or financial relationships that could be construed as a potential conflict of interest.

## Publisher's note

All claims expressed in this article are solely those of the authors and do not necessarily represent those of their affiliated organizations, or those of the publisher, the editors and the reviewers. Any product that may be evaluated in this article, or claim that may be made by its manufacturer, is not guaranteed or endorsed by the publisher.
